# In-House, Fast FDM Prototyping of a Custom Cutting Guide for a Lower-Risk Pediatric Femoral Osteotomy

**DOI:** 10.3390/bioengineering8060071

**Published:** 2021-05-26

**Authors:** Leonardo Frizziero, Gian Maria Santi, Christian Leon-Cardenas, Giampiero Donnici, Alfredo Liverani, Paola Papaleo, Francesca Napolitano, Curzio Pagliari, Giovanni Luigi Di Gennaro, Stefano Stallone, Stefano Stilli, Giovanni Trisolino, Paola Zarantonello

**Affiliations:** 1 Department of Industrial Engineering, Alma Mater Studiorum University of Bologna, 40136 Bologna, Italy; gianmaria.santi2@unibo.it (G.M.S.); christian.leon2@unibo.it (C.L.-C.); giampiero.donnici@unibo.it (G.D.); alfredo.liverani@unibo.it (A.L.); paola.papaleo2@studio.unibo.it (P.P.); francesca.napolitan5@studio.unibo.it (F.N.); cuzio.pagliari@studio.unibo.it (C.P.); 2 IRCCS—Istituto Ortopedico Rizzoli (Rizzoli Orthopaedic Institute), Paediatric Orthopaedics and Traumatology, 40136 Bologna, Italy; giovanniluigi.digennaro@ior.it (G.L.D.G.); stefano.stallone@ior.it (S.S.); stefano.stilli@ior.it (S.S.); giovanni.trisolino@ior.it (G.T.); paola.zarantonello@ior.it (P.Z.)

**Keywords:** 3D printing, orthopedic reproduction model, CT scan, cutting guide, CAD surgery simulation, CASS

## Abstract

Three-dimensional printed custom cutting guides (CCGs) are becoming more and more investigated in medical literature, as a patient-specific approach is often desired and very much needed in today’s surgical practice. Three-dimensional printing applications and computer-aided surgical simulations (CASS) allow for meticulous preoperatory planning and substantial reductions of operating time and risk of human error. However, several limitations seem to slow the large-scale adoption of 3D printed CCGs. CAD designing and 3D printing skills are inevitably needed to develop workflow and address the study; therefore, hospitals are pushed to include third-party collaboration, from highly specialized medical centers to industrial engineering companies, thus increasing the time and cost of labor. The aim of this study was to move towards the feasibility of an in-house, low-cost CCG 3D printing methodology for pediatric orthopedic (PO) surgery. The prototype of a femoral cutting guide was developed for its application at the IOR—Rizzoli Orthopedic Institute of Bologna. The element was printed with an entry-level 3D printer with a high-temperature PLA fiber, whose thermomechanical properties can withstand common steam heat sterilization without bending or losing the original geometry. This methodology allowed for extensive preoperatory planning that would likewise reduce the overall surgery time, whilst reducing the risks related to the intervention.

## 1. Introduction

This research finds its purpose in today’s growing need for engineering and surgical collaboration fueled by the development of three-dimensional (3D) printing technology and medical imaging manipulation-dedicated software. The increasing diffusion of fused deposition modeling (FDM) technology has quickly expanded 3D printing applications in a wide range of scientific fields and engineering applications. FDM technology is a powerful and versatile additive manufacturing method: fast prototyping is easily fulfilled using thermoplastic filaments, which are heated to the melting point and then extruded, layer by layer, to create a three-dimensional object. Relying on affordable investments, open-source software and relatively fast printing processes, FDM technology is taking an essential role in orthopedics and traumatology [1]. 

Computer-aided design (CAD) and 3D printing technology have been shown to successfully upgrade diagnostics, and 3D human anatomy modeling is a powerful support to surgical routines. Through computer-aided tomography–computer-aided design (CAT-CAD), tomographic acquisitions become faithful 3D CAD models of patients’ anatomy; through computer-aided design–computer-aided manufacturing (CAD-CAM), bony models are 3D printed in 1:1 scale and serve as a direct means of evaluation of bone injuries and deformities [2,3,4,5,6]. Through computer-aided surgical simulation (CASS), surgical precision is enhanced, relying on extensive preoperatory planning, thus giving doctors a complete understanding of each specific anatomy. This is particularly useful in clinical cases of complex deformity but has little or no interest in common surgical procedures or emergency surgeries. The cutting edge of surgical simulation currently relies on augmented reality research [7]. CASS has been found helpful in advancing preoperative planning; improving surgical outcomes; reducing blood loss, instrumentation time and extensive surgical exposure [8,9,10,11]; enhancing doctor–patient communication [12] and supporting surgical training [13].

Even though some research has found that computer-aided surgical simulations and 3D printed models are not always accessible due to their high cost and time dissipation [14], it is commonly observed that procedures of computer-aided extensive surgical planning are blatantly successful in reducing the overall operative time and fluoroscopy image acquisition, and have a positive impact on the surgeon’s mindset before the surgery [8,9,10,11,12,13].

With these considerations, it is easy to imagine that designing and printing highly customized surgical tools such as orthopedic cutting guides are possible and strongly demanded.

### 1.1. Custom Cutting Guide (CCG) 

**Figure 1:**
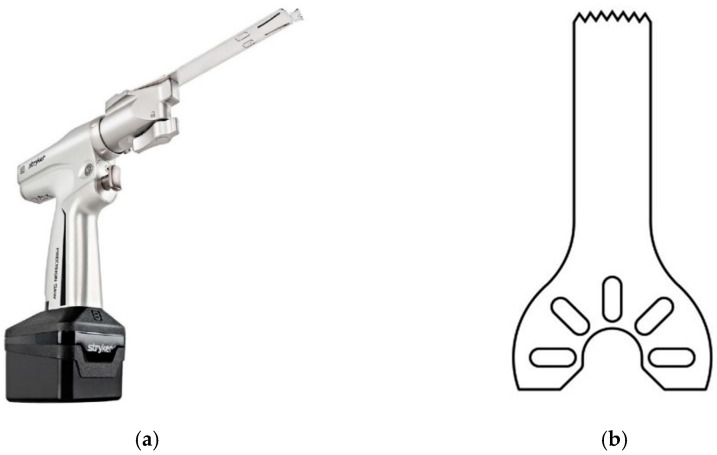
(**a**) Stock photo of a System 8 surgical saw by Stryker. (**b**) Orthopaedic sawblade.

The customized cutting guide (CCG) is a medical device designed to lead the sawblade inside the bone in wedge-resection osteotomies. Surgical procedures of wedge-resection are intended to correct angular bone deformities by subtracting a preplanned cuneus in the zone of maximum curvature. Cuts are performed using an oscillating surgical saw, a specific tool shown in Figure 1. Current surgical practice does not include nonflexible tools to maintain pre-planned angles and section planes during the cut, implying a risk of lower surgical precision as well as an increased risk of infections and blood loss due to the longer operative time. Osteotomies are generally performed “freehand”, bone sections are measured intraoperatively with simple rulers, proper instrumentation (plates, screws, pins etc.) is evaluated in the theater and outcome quality mostly relies on the surgeon’s expertise [15]. CCGs can potentially reduce the complexity of surgeries, so that even younger and less experienced surgeons could perform challenging and risky procedures. Even if large-scale CCG applications are still being discussed, the medical literature is unanimous in acknowledging the immense support given to medical teaching and surgical practice. 

### 1.2. CCGs in Maxillofacial Surgery (MFS)

Several studies and branches of surgery have investigated the feasibility of patient-specific cutting guides, underlining the growing interest in highly customized surgical tools. Maxillofacial surgery routines, which are focused on head, neck, tooth, jaw and oral cavity treatments, are already involving large-scale applications of CCGs and CASS procedures, because the simple relative motion of the maxilla and mandible ease surgical outcome expectations. Extraordinary advances have been pioneered in this direction, allowing for complete in-house production of MFS CCGs through highly dedicated software that enables doctors to carry out surgical simulations and customized device prototyping on their own [16].

Because of the possibility of high customization of tooling and guides that meet the complexity and diversity of the skull, 3D anatomy reconstruction and printing of surgical guides, plates and anatomic models are already a cornerstone in maxillofacial surgery [17,18,19,20,21,22].

### 1.3. CCGs in Total Knee Arthroplasty (TKA)

Total knee arthroplasty (TKA) is a common surgical procedure aimed at pain reduction and improvement of motion of patients with end-stage osteoarthritis. It involves the resection of distal femur and proximal tibia, thus considerably benefiting from CCG applications, as well as general arthroplasty of the lower limbs. In 2012, over 82,000 TKAs were performed worldwide involving CCG applications [23]; it was recently documented that CCG use in TKA procedures helped surgeons reduce the operative time and number of fluoroscopy images taken, and decreased the surgery learning curve, so that doctors with different levels of expertise produced similar positive outcomes [24]. Nonetheless, numerous studies have failed to show advances in terms of improved surgical outcomes [25] and patient satisfaction [23], while others have appreciated the advantages related to CCG implementations [26]. TKA CCGs are already part of biomedical industry production and several companies are designing and selling nylon-printed custom guides. In-house TKA CCG manufacturing is not implemented yet; in fact, biomedical companies design customized cutting guides starting from the patient’s diagnostic acquisitions given by the doctors, and printed devices are meant to receive medical approval before being put into use.

### 1.4. CCGs in Long Bones and Pediatric Surgery

Because of the intricacy of long bones and joint mechanics, and because the impact of soft tissues is yet to be considered in CCG design, it is still challenging to justify large-scale adoption of CCGs in terms of improved surgical outcome. Pediatric orthopedic (PO) applications are still in their infancy as well, since surgical outcome expectations are harder to address in consideration of the ongoing skeletal system growth [27]. Many authors have underlined how CCGs for long bone osteotomies, compared with traditional routines, produced no significant improvement in surgical outcome [25]. The advantages currently lie in the reduction of operative and instrumentation time, blood loss and risk of infection, and the possibility of carrying out complete and extensive preoperatory planning so surgeons can focus on their approach in the theater, therefore enhancing speed and accuracy. The main limitations to large-scale CCG adoption are the cost of labor, the cost of printed objects and collaboration with the mechanical industry or specialized medical centers. The need for designing skills and specialized 3D printing technology confines the majority of such research to university hospitals and departments. 

The present study aimed to give impetus to CCG applications by presenting a low-cost 3D printing and prototyping process that could easily become an in-hospital methodology in the near future. Furthermore, this research focused on prototyping a customized 3D printed orthopedic femoral cutting guide designed for a pediatric application of the IOR—Rizzoli Orthopedic Institute of Bologna. 

## 2. Materials and Methods

### 2.1. Case Study

The study was conducted in accordance with the Declaration of Helsinki, and the protocol was approved by the “Area Vasta Emilia Centro” Ethics Committee (CE-AVEC; project identification code: 3D-MALF—356/2018/Sper/IOR) [28]. 

**Figure 2:**
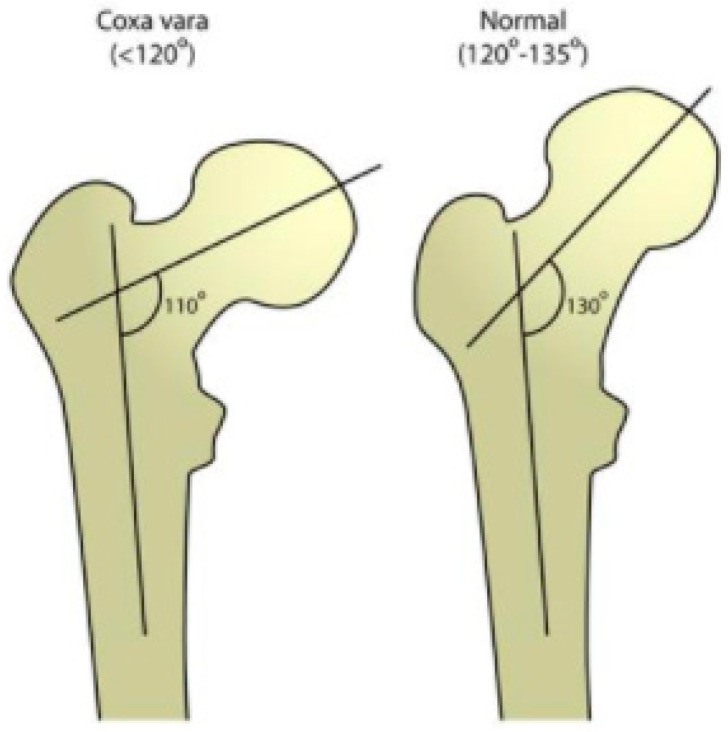
Cervico-femoral angle in cases of coxa vara deformity and normal physiological conditions.

The 5-year-old patient suffered from bilateral coxa vara, an orthopedic condition that is treated through wedge-resection osteotomy. The deformity of the coxa vara is characterized by a reduced cervico-femoral angle (the angle between the neck and shaft of the femur) to less than 120°, which is normally between 135°–145° (Figure 2). Coxa vara is classified into several subtypes, as a congenital, developmental or acquired deformity. The most common cause of coxa vara is either congenital or developmental. The congenital coxa vara is present at birth, caused by an embryonic limb bud abnormality; the developmental coxa vara occurs as an isolated deformity of the proximal femur and it is frequently noticed at the walking age as a leg length difference or an abnormal gait pattern. A less frequent cause is acquired, due to underlying conditions that alter a normal anatomy, including metabolic bone diseases or post-traumatic conditions (due to improper healing of a fracture between the greater and lesser trochanter). The Shepherd’s Crook deformity is a severe form of coxa vara, characterized by a reduction of the neck shaft angle beyond 90°. 

**Figure 3:**
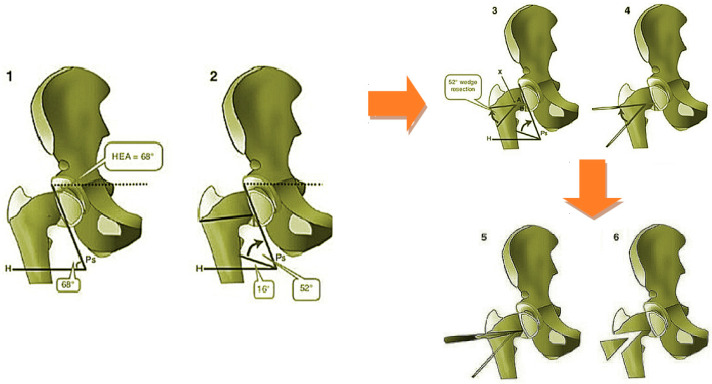
Pauwell’s surgical procedure. Ps is the physeal line, i.e., the bony segment enclosed by the metaphysis and epiphysis of skeletally immature patients. The HEA should be less than 16°; a positive increase in the standard HEA value directly expresses the CCG’s dimension. Here, an HEA of 68° is shown, indicating a varus deformity of (68° − 16°) = 52°. The CCG’s opening angle should cut a bony wedge with a 52° opening angle.

**Figure 4:**
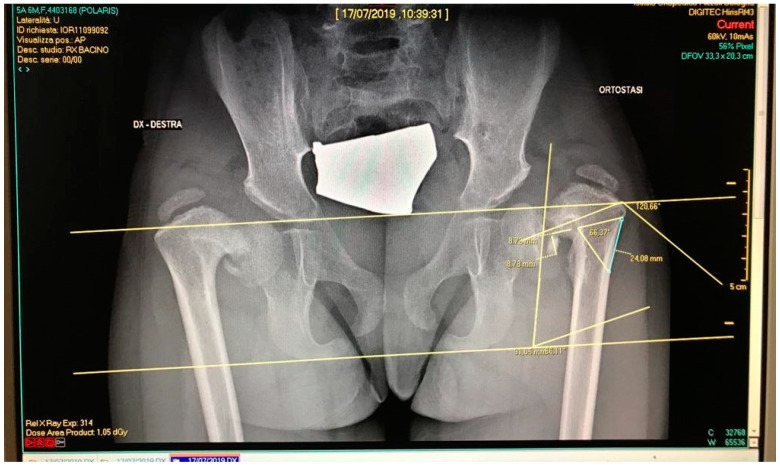
Measurement of the bony wedge on Carestream.

Pauwell’s osteotomy is indicated in severe coxa vara [29]. Preoperatory planning involved calculating the Hilgenreiner epiphyseal angle (HEA) (Figure 3), which should not exceed 16°. An increase of this value implies a varus deformity, measured as the difference between the HEA’s actual value and the standard HEA value (16°). This difference corresponds to the bony wedge opening angle that should be removed in order to achieve a positive surgical outcome. The angle is marked proximally by a horizontally parallel pin under the greater trochanter to the cartilage of the inferomedial femoral neck and distally by an oblique pin in the lateral cortex aimed towards the first pin. The intersection of these 2 pins creates the wedge. Once the wedge has been removed, the osteotomy is completed medially. The angle is closed by pulling the proximal fragment laterally and down, then the distal femur in abduction. Preliminary wedge measurements were carried out following the same procedure on Carestream Vue PACS v11.3.4 (Carestream Health Inc., Rochester, NY, USA) (Figure 4). This software supports doctors in performing linear and angular measurements on diagnostic bidimensional images. Following this procedure, however, the magnitude of measurement error is hardly unremarkable and several studies have underlined how two-dimensional evaluation does not always provide accurate information on complex three-dimensional deformities [25]. Clinical cases of complex deformity, which is defined as occurring in at least two anatomical planes, need extensive preoperatory planning and generally lead to invasive surgeries. Having considered this evidence, it is easy to imagine how the 3D representation of bony structures is changing the way surgeons approach preoperatory planning.

### 2.2. CAM and CAD-CAT

**Figure 5:**
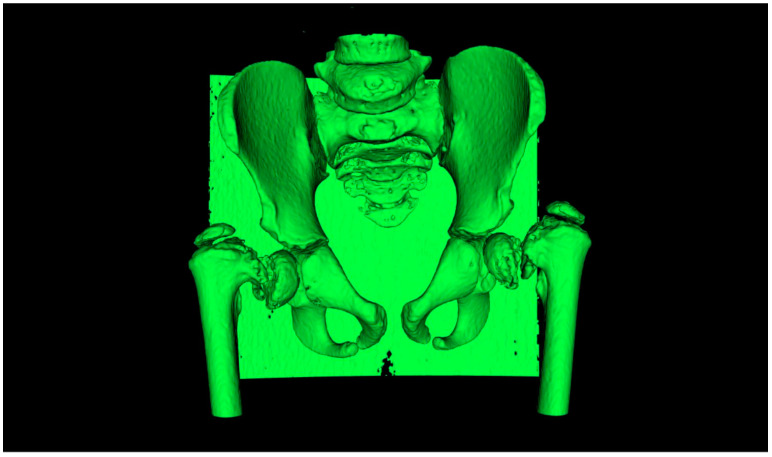
Reproduction of the 3D surface on InVesalius; a custom density range was applied to select the area of interest.

**Figure 6:**
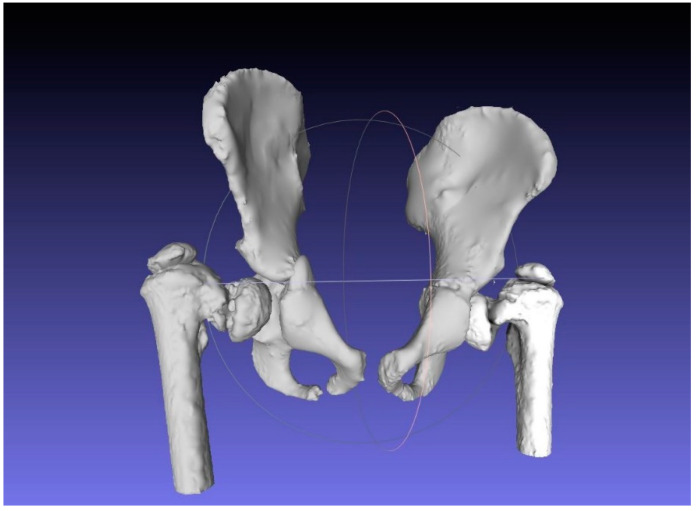
Mesh optimization on MeshLab.

Digital Imaging and Communication in Medicine (DICOM) files acquired by the CT machine were imported to InVesalius v3.1 (CTI: Center for Information Technology “Renato Archer”, Campinas SAO, Brazil, 2017). The first step of the workflow consisted of reproducing the 3D surface of the patient’s skeleton involved in the operation. Because of the patient’s young age and the peculiarity of the proximal femur, which is a particularly spongy bone tissue, a really careful study of the imposed density range was necessary (Figure 5). Bone density range was set between 125 and 440 HU. Three-dimensional reconstructions of both proximal femurs and the pelvis were exported from InVesalius, then imported in Standard Triangulation Language (STL) format and processed through MeshLab v2016.12 (ISTI-CNR “Alessandro Faedo”, Pisa, Italy, 2016) undergoing a process of cleaning and hollowing by subtracting the bone marrow (Figure 6). This aspect was considered, since the interior bony structure is not investigated in such surgical planning, and lighter meshes undergo a very rapid modeling and 3D printing process. Final mesh corrections were performed using another open-source software package, Meshmixer v2017 (Autodesk, San Rafael, CA, USA, 2017). 

### 2.3. CASS and CCG Design

**Figure 7:**
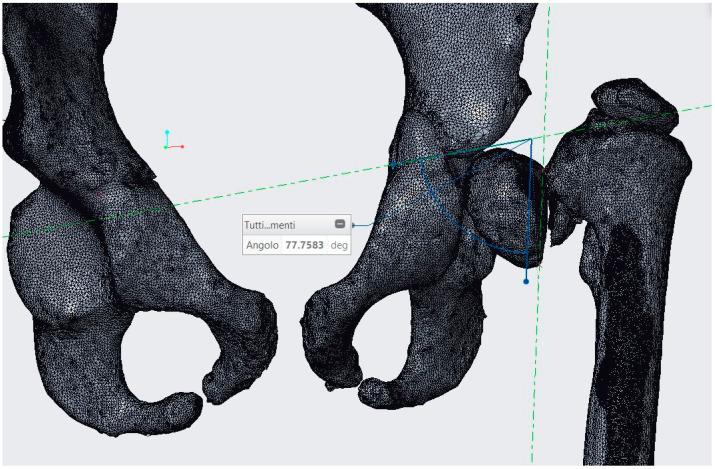
78° varus deformity measured on Creo Parametric.

**Figure 8:**
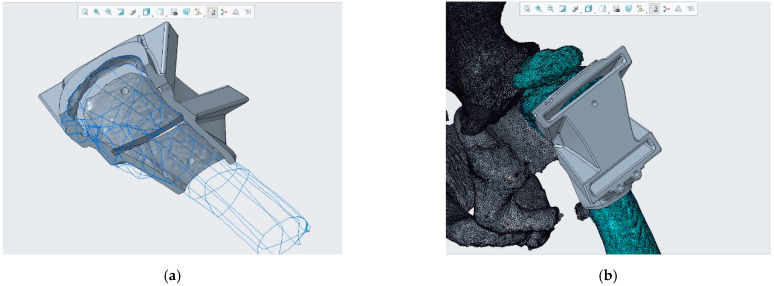
CCG design on Creo Parametric. (**a**) The base of the CCG is extruded on outer cortex; (**b**) slits for the sawblade following previous CASS and holes for steel pins were added.

**Figure 9:**
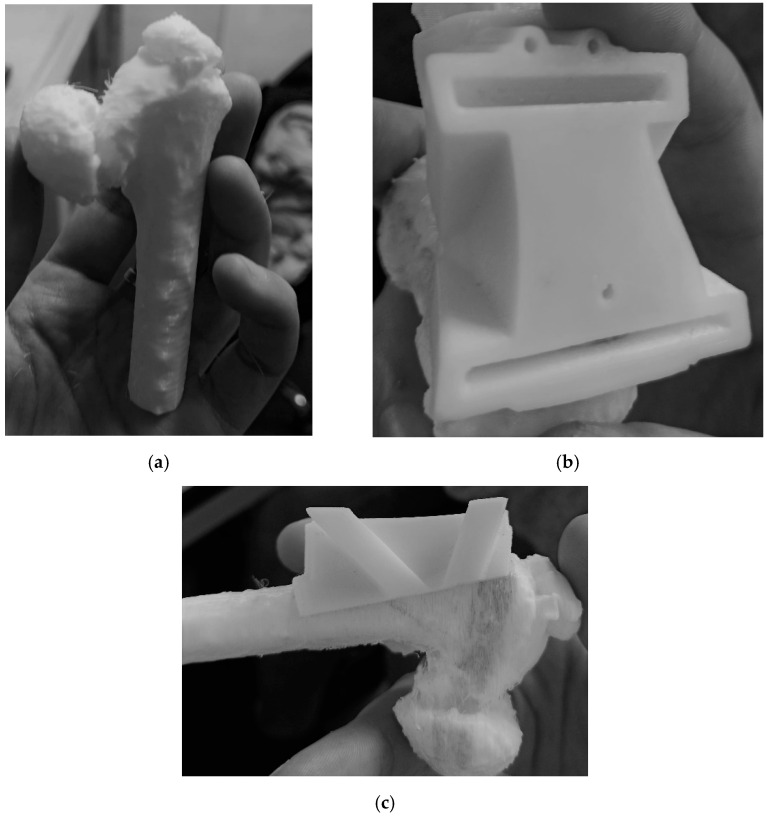
Three-dimensionally printed bony model and CCG: (**a**) 3D printed femur; (**b**) CCG top view; (**c**) CCG on bone model, lateral view.

Following surgical indications, anatomical measurements and reference axes were transferred to the 3D bony model using Creo Parametric (Figure 7). CASS consisted of verifying the geometric measurements on the pelvis and femurs with the purpose of correcting the wedge opening angle and determining the landmarks of the cut. The 3D bone model showed 78° of varus deformity, so HEA = (78° − 16°) = 62°. The Carestream wedge angle value was 66°, so measurements of diagnostic images instead of the diagnostic 3D model produced 4° of angular error. After these measurements had been verified by surgeons, a reverse-engineering CCG designing process was applied. The femoral surface was segmented and extruded so that the internal surface of the CCG would fit perfectly on the patient’s cortical bone (Figure 8a,b). Slits and holes for steel pins were designed along the previously measured cutting planes in order to sustain the sawblade during the cut. In the interest of achieving complete preoperatory planning, both proximal femurs and cutting guides were 3D printed (Figure 9a–c). The artifacts were printed using a low-cost 3D printer (EZT3D model T1) from high-temperature poly-lactic acid (HTPLA) and sterilized in an autoclave for 45 min with 132 °C saturated steam under pressure, which is the industry standard sterilization method, showing no signs of bending or losing geometry at the end of the process. Maintenance of the design geometry was detected by visual inspection and assembly on the 3D printed femoral model with sufficient accuracy. 

**Figure 10:**
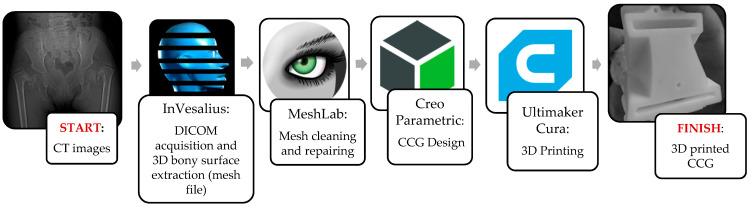
Steps of the workflow.

CAD licenses represent a relevant cost item in CASS and CCG design. This methodology involved the use of open-source software: InVesalius, MeshLab and Ultimaker Cura. CREO Parametric is not open-source software, but offers the opportunity of a limited student version or a full version 30-day trial. This step can also be performed with open-source parametric CAD, such as FreeCAD (DigitalOcean, KiCad Service Group, New York, NY, USA, 2021). A summarized workflow of the procedure from start to finish is shown in Figure 10. The availability of the implemented software is stated in Table 1.

### 2.4. Sterilization of the Polymer-Based Medical Elements

Other CCG osteotomies on the upper and lower limbs were examined and compared, in order to assess the process’s convenience in terms of the chosen material, 3D printing technology and sterilization method (see following Table 1, Table 2, Table 3 and Table 4). The last one is a crucial aspect because 3D printed CCGs need to be properly sterilized while maintaining their mechanical resistance and design geometry, but most FDM polymers are unable to withstand common sterilization techniques. Steam heat sterilization (autoclaving) is the most common and one of the most effective sterilization methods for surgical tools [30]. Furthermore, it has been observed that steam heat sterilization of PLA artifacts is safe and efficient for pathogen elimination [31]. Aggressive steam heat cycles are up to 134 °C, but several FDM polymers such as acrylonitrile butadiene styrene (ABS), polyethylene terephthalate glycol (PETG) and simple polylactic acid (PLA) cannot bear more than 50 °C without significantly losing mechanical properties. Low-temperature sterilization methods are generally applied to these polymers. The medical literature reports ABS CCG being sterilized at 37 °C using Ethylene Oxide (EtO) [26], a toxic gas that needs up to 16 h to complete a cycle due to the mandatory air washing cycles. The same method was applied to simple PLA CCG [32] and to PA2200 (nylon) CCG [33]. Steam sterilization of nylon printed artifacts is still being questioned; however, other research [34] has involved industrial CCGs from biomedical companies that supported aggressive steam cycles. It is important to underline that nylon fibers are commonly printed using a very expensive technology called selective laser sintering (SLS) and, similar to ABS printing fibers, harmful gases are emitted during the process [35]. Process safety is indispensable in order to exclude the cost of specialized personnel and to reduce 3D printer investment. Other authors [36] reported the production of PETG printed CCGs and sterilization using hydrogen peroxide. Generally, this procedure is carried out in a temperature interval of 28–40 °C and the overall time can be up to 8 h. Another remarkable FDM polymer is polyether-ether-ketone (PEEK). It has excellent thermomechanical resistance and the property of biocompatibility, and it is frequently used in the production of medical devices [37]. However, it was not used in this study because it is much more expensive than PLA polymers. This application required the deployment of a low-end FDM machine and affordable materials, biocompatibility, autoclavability and the employment of non-specialized staff. These concerns have encouraged nylon, ABS and PETG to be discarded, and HTPLA to be chosen for its biocompatibility, autoclavability and low-end printing process. In order to assess the advantages of the preferred material, a comparison with similar biocompatible FDM fibers available on the market was carried out; the main aspects are summarized in Table 2.

### 2.5. Main Features of Low-End and High-End FDM 3D Printers 

FDM 3D printers are found to have various characteristics. The price range goes from hundreds (desktop 3D printers) to thousands of Euro (professional 3D printers). However, common features and functions are detectable as follows:

− An hotend, which heats the filament and extrudes it outside the nozzle;
− The printing bed, which can be static or mobile, heated or non-heated;
− A cooling fan;
− The frame, which has to absorb vibrations and guarantee stability during the process;
− The material supply system, which is linked to the hotend through a polytetrafluoroethylene (PFTE) tube, giving the filament a precise path to follow (Bowden), or simply fixed directly on the x-axis (direct drive);
− Rails and engines: rails are found in different constructive solutions. Low-end 3D printers usually use linear bar rails with vertical bearings, which is the most economical but inaccurate configuration because of the friction between the linear rail and the bearing. Another possible constructive solution is through a rubberized wheel on the extruded aluminum rail, which has silent disposition and has a good ratio between cost and efficiency. High-end FDM printers are equipped with linear rails, which represent the most expensive but most accurate solution.

High-end solutions could also include additional heating or flow regulation systems, such as cameras that facilitate monitoring of the process. A heated bed, which is an additional feature, is needed in the 3D printing of polymers that present issues of shrinkage and thermal expansion, such as ABS fibers. PLA fibers do not suffer from thermal distortion; furthermore, it has been documented that very little discrepancy in accuracy occurs when comparing PLA artifacts realized with low- and high-end printers [38]. High-end FDM machines usually present upgraded printing speed and vibration absorption, and the geometrical artifacts’ accuracy is slightly improved as a consequence of the much more rigid and cushioned structure. Therefore, using a low-end FDM 3D printer is a suitable choice for this application. The specific 3D printer that allowed for the realization of the current prototype is a low-end FDM 3D printer with an indicative cost of EUR300. Overall printing time resulted to be 1.5 hours. The printing parameters are summarized in Table 3. 

## 3. Results and Discussion

The CCG here presented is a prototype and was not applied to the medical procedure. Since it was realized in collaboration between a university hospital and an engineering department, the cost of labor was not included in the process. This CCG is meant to undergo resistance tests in order to evaluate mechanical behavior in relation to the possible impact from the sawblade. Final approval from the hospital directors and those responsible for the surgery is required in order to allow the usage of the prototype given in this study in actual surgical practices. Moreover, a source of biocompatible-authorized-grade HTPLA should be obtained in order to get consent for the application. However, it served as a powerful means of conveying medical information through extensive preoperatory planning. Following this path, reductions of the operative time and measurement corrections were possible. A full-scale 3D printed model was used to carry out proper surgical tool evaluation, resulting in a further reduction of intraoperative instrumentation time. The CCG underwent a process of steam heat sterilization that allowed the design geometry to be maintained. 

The aim of this paper was to show an informatic methodology of rapid prototyping meant to ease extensive preoperatory planning and overall time reduction in clinical studies of complex deformity. It is possible to extend this procedure to a wide number of medical treatments that could benefit from the application of highly customized surgical tools. CCG technology embodies an engaging instrument, and large-scale adoption could widely meet medical advances, as patient-specific approaches are more and more investigated, and surgical training is more and more virtually oriented. Nonetheless, a few impediments are delaying its widespread application in surgical procedures. Concerns regarding managing the learning curve of the design skills required, the increased capital costs and third-party involvement often stop the acceptance of these technologies. Hospitals are pushed to search for design and prototyping skills in the industrial world, increasing the costs for healthcare systems and downtimes in preoperatory planning. This research aimed to give impetus to this growing surgical demand by proposing an alternative low-cost in-house CASS and 3D printing methodology. The chosen material, HTPLA, is a PLA fiber with great thermomechanical properties and a relatively low cost (1 kg costs EUR65). Unlike other 3D printable fibers such as ABS and nylon PA-12, it does not emit harmful gases during its extrusion; thus, it is possible to choose an entry-level 3D printer with no closed chamber for the printing. Furthermore, HTPLA-printed artifacts can undergo steam heat sterilization, which is common in hospitals and faster than other methods of sterilization that are often required for 3D printed objects [39]. 

Cost items to consider for CCG design, manufacturing and application are: *production* (in-house or third-party involvement), informatic procedure (software licenses), 3D printing technology and material, and sterilization methodology. No comparison with commercial CCGs was made, since there are no commercial pediatric CCGs. Those that exist are designed for adult patients. As healthcare systems push to reduce cost and time dissipation, in-house production is definitely the ultimate ambition. However, know-how and the required skills limit in-house production mostly to university hospitals and research teams. In-house production requires user-friendly, linear informatic procedures, and even if informatic licenses should potentially be the highest cost item of the overall research, open-source dedicated software is already being used both by doctors and engineers. Materials and the subsequent 3D printing process should be chosen considering biocompatibility, process economy and the sterilization technique. According to the last 10 years of medical literature, medical-grade resin-based CCGs are being replaced by PLA-based CCGs. Medical resin is produced through the SLA (stereolithography) printing process using ultraviolet light to cure photosensitive polymers. The SLA printing process is generally more time- and cost-consuming than FDM technology, because resin artifacts need to be post-processed in order to show good mechanical properties: resin artifacts are generally weaker than PLA objects and need long and complicated post-processing phases. ABS, PETG and simple PLA are easily printable FDM filaments, but their thermomechanical properties prevent steam heat sterilization, thus needing low-temperature sterilization processes that are much longer and less efficient. Nylon objects are printed mostly through SLS technology, which is an expensive and complex procedure, or through high-cost industrial FDM printers (Markforged Mark Two). Sterilization of nylon artifacts is divisive, as some authors are using EtO because aggressive cycles lower its mechanical properties, but several companies recommend sterilization with very short cycles of high-pressure steam heat in a protective bag. HTPLA is FDM printable with a simple desktop 3D printer and can easily bear aggressive steam heat cycles while maintaining the same designed geometry, and, after a short annealing process, its thermo-resistance could be enhanced [40]. It is believed that PLA-based polymers will shortly become a permanent part of medical equipment. 

## 4. Conclusions

A simple workflow on open-source software was revealed here (except for the CCG design with Creo Surfacing), using simple 3D printing technology, low-cost material and rapid sterilization. Extensive surgical planning was easily conducted on the 3D printed bony model, reducing overall surgical and instrumentation time, and enhancing the precision of anatomical measurements. The wedge opening angle was calculated in a 3D parametric environment, enhancing the accuracy of surgical correction. A previous measurement on bidimensional images of 66° was corrected on the 3D bony model, resulting in 62°, showing how angular measurements on bidimensional images produce uncertain values that could potentially lower the surgical outcome quality. There are differences between 2D and 3D software used for element measuring, which can lead to unknown problems during surgery and the overall result. A HTPLA-printed CCG was produced and sterilized aggressively, maintaining its mechanical properties and design geometry.

## Figures and Tables

**Table 1:** Availability of implemented software packages.

Software	Availability
InVesalius	Open-source
MeshLab	Open-source
Creo Parametric	Student license// 30-day free trial
Ultimaker Cura	Open-source

**Table 2:** Selection criteria of available biocompatible FDM polymers.

Material	Autoclavability	3D Printing Capability	Cost (EUR/kg)
HTPLA (ProtoPasta)	Yes	Low-end	68
Medical ABS (FiloAlfa)	No	Heated bed Closed chamber Gas emissions	56
Antibacterial PLA (FiberForce)	No	Low-end	73
nGen Flex (ColorFabb)	No	Heated bed Post-processing (cooling phase)	62
PLA Bioflex^®^ (FiloAlfa)	No	Low-end	60
PEEK	Yes	Heated bed Post-processing (mechanical resistance enhancement)	400

**Table 3:** Printing parameters.

Parameters	Values
Nozzle temperature (°C)	210
Printing speed (mm/s)	25–45
Nozzle diameter (mm)	0.4

**Table 4:** CCG design comparisons in the literature.

Ref.	Production	Informatic Procedure	Material	Sterilization Method	3D Printing Technology
This procedure	In-house	Invesalius + MeshLab (Meshmixer) + PTC Creo	HTPLA	Steam-heat	FDM
[29]	In-house	Orthoview + OsiriX + Meshmixer	ABS	Ethylene oxide	FDM
[32]	In-house	Orthoview + Meshmixer	Acrylate resin	*	FDM
[33]	In-house	Mimics	Nylon	Ethylene oxide	SLS
[34]	PROPHECY	*	Nylon	Steam heat	SLS
[35]	In-house	OsiriX + Netfabb + Fusion 360	PETG	Hydrogen peroxide	FDM

* Not mentioned in the paper.

## Data Availability

Please refer to suggested Data Availability Statements in section “MDPI Research Data Policies” at https://www.mdpi.com/ethics.
